# Synthesis Amphiphilic One-Handed Helical Ladder Polymers with Circularly Polarized Luminescence

**DOI:** 10.3390/molecules30122606

**Published:** 2025-06-16

**Authors:** Ziheng Pan, Wei Zheng

**Affiliations:** Interdisciplinary Materials Research Center, School of Materials Science and Engineering, Tongji University, Shanghai 201804, China; 2230644@tongji.edu.cn

**Keywords:** chiral, helical ladder polymers, circularly polarized luminescence

## Abstract

Helical ladder polymers attract attention because of their well-defined, one-handed helical ladder structures and unique properties, which differ from precursor polymers that have random-coil conformations. However, the synthesis of helical ladder polymers is difficult and inhibits their functions and applications. In this study, we reported the synthesis of amphiphilic optically active 2,2′-tethered binaphthyl-embedded helical ladder polymers carrying hydrophilic oligo (ethylene glycol) (OEG) as side chains through quantitative and chemoselective acid-promoted intramolecular cyclization of random-coil precursor polymers. The obtained helical ladder polymers exhibited dramatic circular dichroism (CD) and circularly polarized luminescence (CPL) enhancement. Moreover, we further established a circularly polarized fluorescence-energy transfer (CPF-ET) strategy in which the helical ladder polymers work as a donor, emitting circularly polarized fluorescence to excite an achiral fluorophore (coumarin-6) as the acceptor, producing green CPL with luminescence dissymmetry factor (2.5 × 10^−4^).

## 1. Introduction

Helical polymers, inspired by the elegant and intricate helical architectures found in biomacromolecules such as DNA and proteins, have become a focal point of modern polymer science [1]. In biological systems, these naturally occurring helices play essential roles, from genetic information storage and transmission to maintaining structural integrity and enabling biochemical functions [2]. Motivated by the complexity and functionality of these natural systems, researchers have devoted extensive effort to designing and synthesizing artificial helical polymers [3]. These polymers, characterized by their controlled helical conformations, exhibit unique physical, chemical, and optical properties, making them highly promising for applications in the chirality stationary phase [4], information encryption [5] and biomedicine [6].

Recently, helical ladder polymers, as a unique class of helical polymeric architectures possessing both ladder and helix geometries, have received much attention. The rigidity of the ladder framework imparts structural stability and thermal resilience, while the helical arrangement introduces chirality and opportunities for tunable optical and electronic properties [7]. Recent advancements in synthetic methodologies have facilitated the development of these polymers with precise control over their structures, enabling the systematic exploration of their unique properties [8]. Scherf et al. [9] synthesized and characterized the first successful helical ladder polymer in 2006. They first obtained the rigid main chain by microwave-assisted Suzuki coupling through the use of binaphthyl building blocks. After that, the helix-like ladder polymer was obtained by Friedel–Crafts intramolecular electrophilic cyclization, triggered by boron trifluoride etherate. Another successful case of helical ladder polymer was reported in 2019, when Ikai et al. [10] constructed a new family of triptycene-based helical ladder polymer with a well-defined, one-handed helical geometry through polymerization, followed by an efficient ladderization approach. Subsequently, the same group successfully realized a series of defect-free spirobiindane, or binaphthyl-embedded helical ladder polymers, by the quantitative and chemoselective acid-promoted alkyne benzannulations, assisted by introducing 2,6-dimethyl substituents on the phenyl pendant groups, some of which can be applied to chromatographic enantioseparation as chiral stationary phases for high-performance liquid chromatography due to its unique π-electron-rich cylindrical helical cavity [11]. Very recently, Meskers et al. [12] prepared a new spirobifluorene-based, one-handed, rectangular-like ladder polymer and an in-depth study of physical excited states of chromophores arranged within the helical architecture. However, despite significant advancements in this field, most helical ladder polymers exhibit rigid helical backbones and poor water solubility, which inhibits their functions and applications [13].

Circularly polarized luminescence (CPL) materials are drawing ever-increasing interest in both materials science and chirality-associated applications for their significant potential in three-dimensional optical displays, optical storage devices [14] and photoelectric devices [15]. To date, numerous CPL-active materials have been constructed from metal–organic molecules [16], organic supramolecules [17] and polymers [18]. Gong et al. [19] reported a Cu-based core complexed with carbene–metal–amide (CMA) and an acridine ligand, exhibiting pronounced CPL signals. The chiral properties arise from the restricted ligand–ligand rotational freedom of the CMA enantiomers in the aggregated state, leading to dissymmetry factor values as high as 2.7 × 10^−2^. Moreover, the metal core has been shown to facilitate CPL expression under a magnetic field [20]. In addition, new properties such as thermally activated delayed fluorescence [21] (TADF) and circularly polarized electroluminescence [22] (CPEL) are explored in the device, which demonstrates that this kind of molecule behaves well, as the CPL materials like organic light-emitting diodes. Compared with the chirality of small molecules, the helical-ladder polymer-based CPL materials have promising and diverse CPL properties due to their ability to restrict the rotation of repeating units by covalent bonds. Nevertheless, research on color-tunable CPL emissions remains limited [23], being primarily hindered by the scarcity of robust and straightforward synthetic methodologies. Advances in supramolecular chemistry now enable chiral self-assembly/co-assembly systems to synergistically harness chiral transfer and intermolecular Förster resonance energy transfer (FRET), thereby amplifying induced CPL signals in achiral dyes [24]. Deng’s group has prepared varieties of helical polyacetylene-based CPL materials through the chiral induction of chiral helical polymers to fluorescent dyes [25]. Inspired by this type of material, combining helical ladder polymers and achiral small molecular fluorophores may improve the CPL’s performance and its applications.

Herein, we present the successful combination of Suzuki–Miyaura coupling polymerization and acid-promoted alkyne benzannulations to prepare a new family of optically active, amphiphilic, one-handed helical ladder polymers, which are composed of binaphthyl-embedded helical-ladder polymer main chains and water-soluble OEG side chains. The obtained amphiphilic helical ladder polymers exhibited a dramatic ladderization-induced enhancement in CD and CPL due to their well-defined, one-handed helical geometry. To achieve color-tunable CPL, the fluorescent dye coumarin-6 was doped for FRET and chiral transfer in the helical ladder polymers in mixed solutions. The developed system offers significant advantages over existing color-tunable CPL materials, providing a new strategy for the design of advanced color-tunable CPL materials.

## 2. Results and Discussion

### 2.1. Synthesis of the Amphiphilic One-Handed Helical Ladder Polymers

**Scheme 1:**
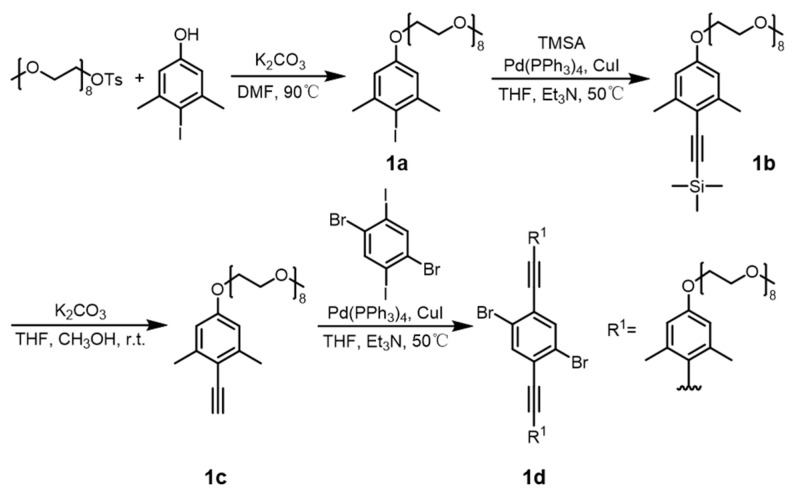
Synthesis of the achiral monomer **1d**.

**Scheme 2:**
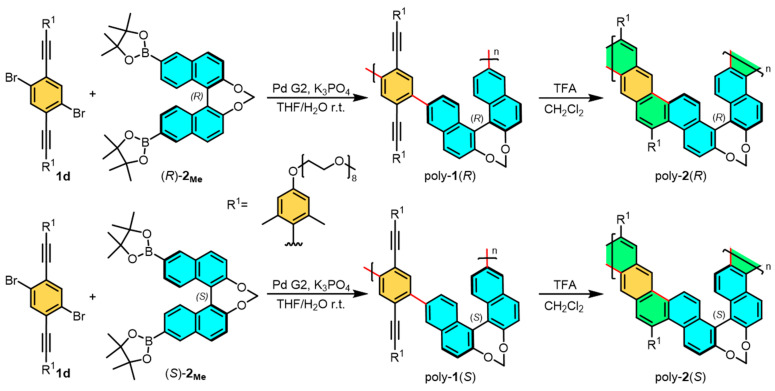
Synthesis of poly-**1**(*R/S*) and poly-**2**(*R/S*).

**Figure 1:**
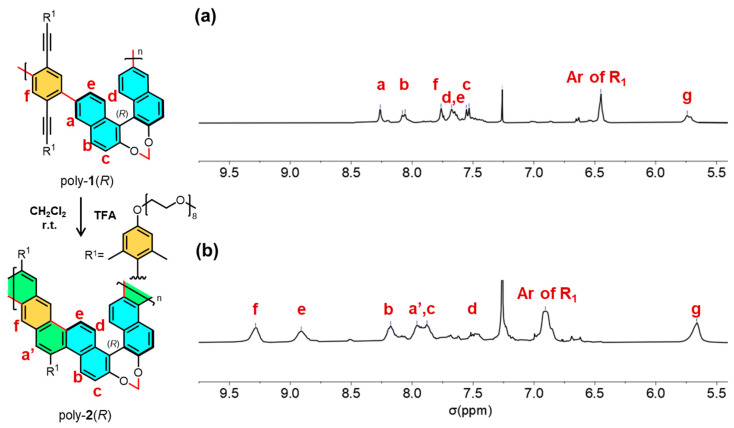
Partial ^1^H NMR spectrum (400 MHz, 298 K, CDCl_3_) of poly-**1**(*R*) (**a**) and poly-**2**(*R*) (**b**). (peaks ‘a to f’ of 1H spectra was attributed to the H-group in the polymer structure shown in the left).

In 2024, Ikai et al. [26] achieved the first synthesis of a series of 2,2′-tethered binaphthyl-embedded helical ladder polymers functionalized with hydrophobic-branched alkyl side chains, effectively suppressing solution-phase aggregation of the helical frameworks. Inspired by this work, we strategically replaced the hydrophobic side chains of helical ladder polymers with water-soluble OEG chains(see synthetic procedure in Scheme 1), thereby modulating the polymer hydrophilicity, and further investigated their co-assembly dynamics and aggregation behavior. The synthesis of the one-handed helical ladder framework is illustrated schematically in Scheme 2. Initially, achiral di-Br monomer **1d** functionalized with water-soluble OEG chains was synthesized through a multi-step protocol, establishing the foundational building block for subsequent polymerization. The precursor polymers, poly-**1**(*R*) and poly-**1**(*S*), were synthesized by the copolymerization of (*R*) or (*S*) 2,2′-methylenedioxy-tethered-1,1′-binaphthyl-based diboronic acid ester with p-dibromobenzene derivative **1d** via Suzuki–Miyaura coupling. Subsequently, the poly-**1**(*R*) and poly-**1**(*S*) were treated with trifluoroacetic acid (TFA) to transform their random-coil conformations to the rigid-rod, one-handed helical ladder structures poly-**2**(*R*) and poly-**2**(*S*), respectively. It is shown in Figure 1 that the resonance peaks in the ^1^H NMR spectrum of poly-**1**(*R*) and poly-**2**(*R*), where the vanishment of the peak at 8.26 and the new single, well-resolved resonance peak at 7.96 indicated the formation of the ladder framework (peak a in Figure 1a and a’ in Figure 1b, respectively). Moreover, the characteristic resonance signals observed at around 9.01 and 8.85 ppm (peaks e and f, respectively) in Figure 1b moved to low field in contrast to those in Figure 1a (7.76 and 7.53 ppm), suggesting the formation of dibenzo[a,h]anthracene ring systems. It is worth pointing out that all the proton resonances of poly-**2**(*R*) and poly-**2**(*S*) in the aromatic zone is consistent with previous work [26]. In addition, the broad peak near 3.5 ppm belongs to the characteristic peak of OEG, which indicates the successful synthesis of OEG-functionalized ladder polymers. The consumption of ethynyl groups in poly-**1**(*R*) was confirmed by IR spectrum measurements, in which the C≡C stretching band at 2200 cm^−1^ observed for poly-**1**(*R*) was not detectable, which reflected high reaction conversion during the benzannulation process (Figure S1). The number-average molar mass (*M*_n_) of the resulting poly-**2**(*R*) and poly-**2**(*S*) was estimated to be 2.8 × 10^4^ (*M*_w_/*M*_n_ = 1.8) by size-exclusion chromatography (SEC). The ladder polymer exhibited good solubility in common organic solvents such as dichloromethane (CH_2_Cl_2_), tetrahydrofuran (THF) and were partially soluble in water (H_2_O).

### 2.2. Chiroptical Properties of Amphiphilic One-Handed Helical Ladder Polymers

The absorption spectrum of poly-**1**(*R*) in CH_2_Cl_2_ showed a main single maximum at 320 nm and a broad peak around 355 nm. After ladderization, the poly-**2**(*R*) absorption maximum was red-shifted from 14 nm up to 334 nm compared with the single-stranded precursor, poly-**1**(*R*). The red shift of the maximum absorption peak originated from the acid-catalyzed cyclization reaction, which extended the π-conjugation in the polymer backbone [27] (Figure S2a). Meanwhile, the broad absorption band at approximately 360 nm originated from the extended conjugated linkage between the binaphthyl and **1d** groups. This band diminished upon replacement of the connecting C–C bond with a ladder structure. Additionally, the absorption shoulder appearing between 375 and 445 nm was attributed to the α-band of the fully fused-ring molecular backbone [28].

In the photoluminescence (PL) spectrum of poly-**1**(*R*) and poly-**2**(*R*) in CH_2_Cl_2_, the emission maximum was also shifted to higher wavelengths at 432 nm upon ladderization, which led to a characteristic bluish emission with a fluorescence quantum yield (Φ_F_) of 13% (Figure S2b). In the solid state, the PL spectra of poly-**2**(*R*) extended into the visible light region, and all peaks showed a distinct red shift. The maximum peak was red-shifted from 35 nm up to 495 nm. This changed the emission color of poly-**2**(*R*) from blue (in CH_2_Cl_2_) to yellowish in the solid state, with a fluorescence quantum yield (Φ_F_) of 5% (Figure S3). The spectral differences of HLPs in the solution and the solid state could be attributed to the changed planarization of the π-system; that is to say, the ladderization strongly affected the electronic properties of the polymers [27].

**Figure 2:**
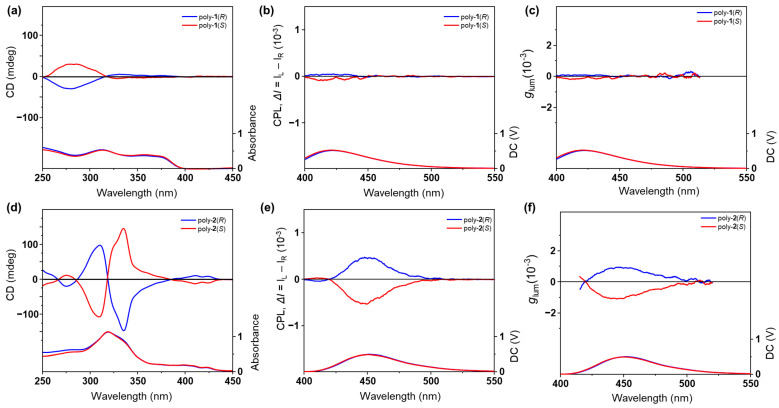
CD-absorption spectra of poly-**1**(*R*) and poly-**1**(*S*) (**a**) and poly-**2**(*R*) and poly-**2**(*S*) (**d**); CPL-DC spectra of poly-**1**(*R*) and poly-**1**(*S*) (**b**) and poly-**2**(*R*) and poly-**2**(*S*) (**e**); and plots of luminescence dissymmetry factors (*g*_lum_)-DC of poly-**1**(*R*) and poly-**1**(*S*) (**c**) and poly-**2**(*R*) and poly-**2**(*S*) (**f**) in CH_2_Cl_2_. The *g*_lum_ values are defined as 2(I_L_ − I_R_)/(I_L_ + I_R_), where I_L_ and I_R_ are the PL intensities of the left-and right-handed circularly polarized lights, respectively (c = 1 × 10^−4^, CH_2_Cl_2_, λ_ex_ = 330 nm).

Circular dichroism (CD) and the absorption spectrum of poly-**1**(*R/S*) and poly-**2**(*R/S*) in CH_2_Cl_2_ are presented in Figure 2a,d. As shown in Figure 2d, poly-**2**(*R*) and poly-**2**(*S*) exhibited intense mirror-image CD signals in the main-chain absorption region (300–400 nm), with a notable bathochromic shift compared with their precursor polymers, poly-**1**(*R*/*S*) (Figure 2a,d). The CD intensity of poly-**2**(*R/S*) exhibited a clear enhancement, in which the |*g*_abs_| value archived 5.17 × 10^−3^ at around 340 nm, which is contrary to that of 0.38 × 10^−3^ at the same wavelength (Figure S4). Poly-**2**(*R/S*) exhibited clear mirror-image CPL signals (|*g*_lum_|= 0.93 × 10^−3^ at 450 nm) (Figure 2e,f) in contrast to poly-**1**(*R/S*) (obviously CPL-inactive in Figure 2b,c). Notably, a similar CPL enhancement could be observed in the solid state; poly-**2**(*R/S*) also exhibited a clear mirror-image CPL signal at their emission range, contrary to the CPL-inactive poly-**2**(*R/S*) (Figure S5). The significantly enhanced CD/CPL intensities of poly-**2**(*R/S*) have been attributed to an interchain exited-state energy transfer to the defect centers. The spectral changes during ladderization indicate that the planarization of the π-system distinctly affects the electronic properties of the polymers. Furthermore, poly-**2**(*R*) displayed identical CD signals across solvents of varying polarity, such as in THF and CH_2_Cl_2_ (Figure S6). Collectively, these findings robustly demonstrate that the incorporation of hydrophilic side chains preserves the structural integrity of the helical ladder polymer’s rigid backbone.

### 2.3. Construction and Optical Properties of Color-Tunable CPL Emission Systems Based on Amphiphilic One-Handed Helical Ladder Polymers

**Figure 3:**
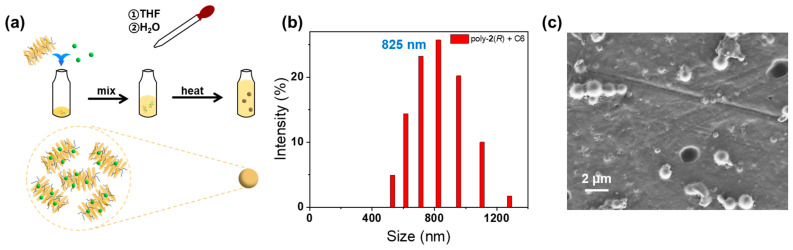
Scheme of the self-assembly of poly-**2**(*R*) with coumarin-6 in THF/H_2_O (3:7, *v*/*v*) (**a**), DLS (**b**) and the SEM image of poly-**2**(*R*) (*c*_poly-**2**(*R*/*S*)_ = 1 × 10^−4^ mol/L according to the repeating units of polymer, *c*_C6_ = 2 × 10^−5^ mol/L) (**c**).

To investigate the polymer’s assembly behavior and chirality transfer effect, we introduced an achiral dye as the acceptor and employed THF/H_2_O as the mixed solvent system. The mixed concentrated solution was supplemented with THF and H_2_O, then subjected to heating, annealing and aging for 2 days to promote aggregate formation through polymer/solvent interactions (Figure 3a). Prior to this, to examine how solvent polarity and interactions govern the chiroptical properties, we dissolved the polymer in varying THF/H_2_O ratios while maintaining a constant polymer concentration. CPL spectra revealed a gradual decline in signal intensity with increasing H_2_O fraction (Figure S7a). Concomitantly, CD spectra exhibited an intensity peak at the THF/H_2_O ratio of (*v*/*v*, 3:7) (Figure S7b). Consequently, THF/H_2_O (*v*/*v*, 3:7) was selected as the optimal condition for polymer assembly. SEM images illustrated that the aggregates take a sphere conformation in the mixed solvent (Figure 3c). The DLS testified that the average dynamic size of poly-**2**(*R*) was mainly distributed around 825 nm, which is consistent with the SEM images (Figure 3b).

**Figure 4:**
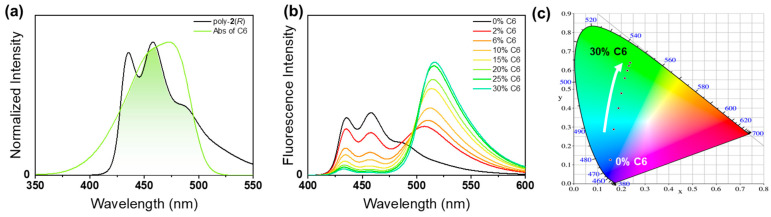
Normalized absorption of coumarin-6 and the PL spectrum of poly-**2**(*R*/*S*) (**a**), fluorescence spectra variations (**b**), and CIE coordinate changes (**c**). (c = 1 × 10^−4^ mol/L. λ_ex_ = 330 nm, in THF/H_2_O, *v*/*v*, 3:7).

Further research on color-tunable CPL emission and chirality transfer were realized through FRET by varying the donor/acceptor ratio in the donor–acceptor (D–A) system. The principles of FRET can be delineated into two parts. First, the donor chromophore in its excited state transfers energy to the acceptor molecule via dipole–dipole coupling in space. The excited acceptor then returns to its ground state by losing energy through photon emission. Meanwhile, the ground-state donor and the acceptor molecules are energetically coupled [29]. The donor’s excitation energy was partially dissipated as self-fluorescence, with the remainder transferred to the acceptor to produce new emissions. For Förster resonance energy transfer (FRET) to occur, two key conditions must be satisfied: (1) the donor (host) and acceptor (guest) molecules must be in close proximity, typically within 100 nm; and (2) there must be substantial spectral overlap between the emission spectrum of the donor and the absorption spectrum of the acceptor. The greater this overlap, the higher the theoretical FRET efficiency [25]. Considering the attractive photophysical and chiroptical properties of the resultant amphiphilic one-handed helical ladder polymers, the further construction of color-tunable CPL emission systems was then performed. Based on the photophysical properties of these helical ladder polymers, coumarin-6 was selected as the fluorescence energy acceptor because its absorption band at around 475 nm obviously overlapped with the emission band of the poly-**2**(*R*) at around 460 nm in THF/H_2_O (*v*/*v*, 3:7) (Figure 4a). As shown in Figure 4b, it was observed that the fluorescence intensity ascribed to the poly-**2**(*R*) donor at 460 nm gradually decreased with the increase in coumarin-6 upon excitation at 330 nm, while a new emission band at around 525 nm derived from coumarin-6 appeared, and the emission color of the solution changed from blue to green under a UV light of 365 nm, showing a typical one-step energy transfer process (Figure 4b). The fluorescence variations, or the CIE (Commission Internationale de l’E’ clairage) coordinate changes, are collected in Figure 4c. To further confirm the energy transfer processes in the supramolecular co-assembly system, the fluorescence lifetime at 460 nm decreased from 7.31 ns to 5.06 ns upon doping of 20% coumarin-6, with an energy transfer efficiency (Φ_ET_) value of 30.8% (Figure S8b), which fully indicated that an effective energy transfer process occurred between poly-**2**(*R*) and coumarin-6.

**Figure 5:**
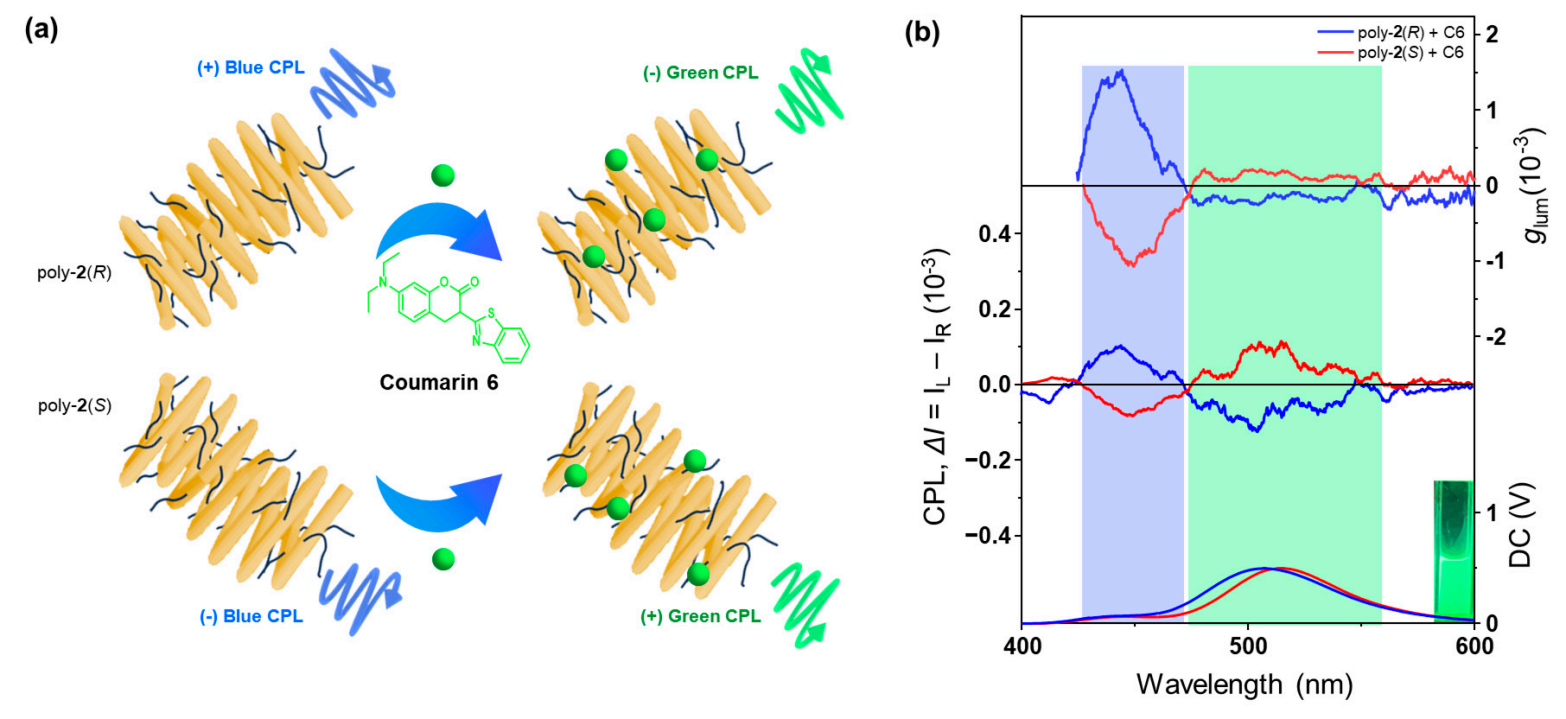
Schematic illustration of the construction and optical properties of color-tunable CPL emission systems based on amphiphilic one-handed helical ladder polymers (**a**), and CPL-*g*_lum_-DC spectra of poly-**2**(*R*/*S*) with coumarin-6 (in THF/H_2_O, *v*/*v*, 3:7, *c*_poly-**2**(*R*/*S*)_ = 1 × 10^−4^ mol/L according to the repeating units of polymer, *c*_C6_ = 2 × 10^−5^ mol/L) (**b**).

The chirality transfer from the helical ladder polymer poly-**2**(*R/S*) to coumarin-6 in the supramolecular co-assembly systems was further explored. Because of the helical chirality of the helical ladder polymers, the CD spectra were obtained for all these co-assembly systems. However, we did not observe obvious chirality transmission signals from the helical ladder polymers to coumarin-6 in the CD spectra with the increasing ratio of C6 (Figure S8a). In contrast, a slight enhancement in absorption in the range of 500–550 nm could be observed in Figure S8a, which is consistent with the absorption of C6 [30]. Considering the fact that these co-assembly systems displayed strong fluorescence in the aggregate state, their CPL responses were attributed to the excited state chirality, which was then investigated. As shown in Figure 5b, under the excitation of 330 nm light, the co-assembly systems exhibited inverted CPL emissions, which were attributed to the acceptor coumarin-6 units at their maximum emission wavelength of 525 nm with a luminescence dissymmetry factor |g_lum_| value of 2.5 × 10^−4^; this was in good agreement with changes in their fluorescence spectra, which indicated the effective FRET of circularly polarized fluorescence from the helical ladder polymer coumarin-6 and was consistent with the analysis of the fluorescence energy transfer. Compared with the CPL spectra of poly-**2**(*R/S*) in CH_2_Cl_2_ (Figure 2) and in THF/H_2_O (Figure S7), the inverted CPL originated from the chirality transfer instead of the polymer itself. It was proposed that in good solvents, the relatively low concentrations of both the chiral small-molecule fluorophores and HLPs made it difficult for them to form the strong noncovalent interactions that are essential for chirality transfer. Upon adding H_2_O, however, these species aggregated into a more compact structure that fulfilled the requirements for FRET [25]. Because the C6 molecules aggregate in a manner aligned with the helical direction of the HLPs, facilitated by hydrogen bonding and π–π interactions between the donor and acceptor, both chirality transfer and energy transfer were thus achieved. According to the reported the chiral and CPL co-assembly system, the achiral dye could capture the supramolecular chirality and collect the circularly polarized energy in the chiral assembly system [31]. Simultaneous inversion of the dissymmetric effect and CPL transfer occurred with the energy transfer, supported by synchronous spectral changes in the CPL-DC profiles of Figure 5b. On the other side, the curve shift was quite undistinguished in the absorbance spectrum in Figure S8a (or could be only observed in the zoomed-in figure), which made it difficult for us to track the change in the CD signals [32]. We could only infer that there would be a slight shift in the CD curve in the absorbance of C6 because the chirality transfer was clearly observed in the CPL-DC spectra. Thus, we realized the emission of CPL from blue to green light by utilizing the FRET effect (Figure 5a).

## 3. Instruments and Materials

### 3.1. Instruments

The ^1^H NMR spectra were recorded on 300 MHz and 400 MHz Bruker (^1^H: 300 MHz and 400 MHz) at 298 K, while the ^13^C NMR spectrum was recorded on 400 MHz Bruker (^13^C: 101 MHz) at 298 K. IR spectrum were recorded on a INVENIO (Bruker Daltonics, Bremen, Germany). The size exclusion chromatography (SEC) measurements were performed with a SHIMADZU liquid chromatograph equipped with a column of the same coordinates, using THF as the flowing phase and a SHIMADZU RID-20A multi-wavelength UV-vis detector (SHIMADZU, Kyoto, Japan). UV-vis absorption (UV-vis) measurements were performed on an Agilent Cary 3500 spectrometer (Agilent Tech, Palo Alto, CA, USA) and were scanned at a rate of 1500 nm/min with a 1.0 nm interval and a 2.0 nm spectrum bandwidth, using a 10 mm quartz cuvette. The circular dichroism (CD) spectrum were obtained in a 1.0- or 10-mm quartz cell using a JASCO J-1700 CD spectrometer (JASCO, Tokyo, Japan). CD spectra of the polymers were recorded in the UV-vis region (250–650 nm). The concentrations of the polymers were calculated based on the monomer units. The HR-MS spectra were recorded by atmospheric pressure chemical ionization (APCI) mass spectrum using Bruker solanX 70 FT-MS (Bruker Daltonics, Bremen, Germany). The photoluminescence (PL) spectra were recorded on an F-4700 FL Spectrophotometer (Hitachi, Tokyo, Japan). The excitation wave of light was set at 330 nm. A scanning rate of 1200 nm min^–1^, an excitation slit width of 5000 μm and a monitoring slit width of 5000 μm were employed. The circularly polarized luminescence (CPL) spectra in the solution were recorded at room temperature on a JASCO CPL-300 (JASCO, Tokyo Japan) with a 10-mm quartz cell. A scanning rate of 100 nm min^–1^, an excitation bandwidth of 30 mm, a monitoring bandwidth of 30 mm, a response time of 4 seconds and 4 repetitions of accumulations were employed. The dynamic light scattering (DLS) data was measured on a Malvern Zetasizer Nano ZS90 light scattering apparatus (Malvern Instruments, Malvern, UK) with a He-Ne laser (633 nm, 4 mW). Scanning Electron Microscopy (SEM) was performed on a ZEISS Gemini instrument (Oberkochen, Germany) at an accelerating voltage of 1 kV and a working distance (WD) of 5 mm.

### 3.2. Materials

Chiral ligands were purchased from Daicel Chiral Technologies Co. (Tokyo, Japan) and used without further treatment. Other starting materials were purchased from Energy Chemical Reagent Co. (Shanghai, China), Adamas (Shanghai, China) and were used as received. All solutions were purchased with safe-dry and safe-seal and degassed with nitrogen for 30 minutes. 

## 4. Synthetic Procedure

**Synthesis of 1a.** The precursor **EG8-Ts** used in the reaction was synthesized according to the previously published reference [33]. To a mixture of **EG8-Ts** (5 g, 8.8 mmol), potassium carbonate (4.4 g, 32 mmol), 4-iodo-3,5-dimethylphenol (2 g, 8 mmol) was dissolved in DMF (10 mL). The mixture was stirred at 90 °C for 24 h. The mixture was diluted with mixed solution (EtOAc: hexane = 4:1, *v*/*v*) and then dried over Na_2_SO_4_. The solvent was removed under reduced pressure, and the crude product was purified by silica gel chromatography using a CH_2_Cl_2_/CH_3_OH mixture (40/1, *v*/*v*) as the eluent to present the desired product as a light-yellow oil (4.4 g, 90% yield). ^1^H NMR (300 MHz, CDCl_3_, 25 °C): *δ* 6.67 (s, 2H), 4.08 (s, 2H), 3.82 (s, 2H), 3.73–3.62 (m, 28H), 3.54 (s, 2H), 3.37 (s, 3H), 2.42 (s, 6H). **Synthesis of 1b.** To a mixture of **1a** (1.4 g, 2.3 mmol), CuI (21 mg, 0.1 mmol) and tetra(triphenylphosphine)palladium (Pd(PPh_3_)_4_) (52 mg, 0.05 mmol), in a degassed THF/Et_3_N mixture (5/2, *v*/*v*; 14 mL), was added along with TMSA, dropwise (0.8 mL, 5.7 mmol). The mixture was stirred at 50 °C for 12 h. After evaporating the solvents, the residue was diluted with CH_2_Cl_2_, washed with brine and water and then dried over Na_2_SO_4_. The solvent was removed under reduced pressure, and the crude product was purified by silica gel chromatography using CH_2_Cl_2_/CH_3_OH mixture (40/1, *v*/*v*) as the eluent to present the desired product as a dark yellow oil (1.2 g, 90% yield). ^1^H NMR (300 MHz, CDCl_3_, 25 °C): *δ* 6.58 (s, 2H), 4.09 (dd, *J* = 5.6, 4.0 Hz, 2H), 3.82 (t, *J* = 4.9 Hz, 2H), 3.64 (d, *J* =3.2 Hz, 28H), 3.57–3.51 (m, 3H), 3.37 (s, 3H), 2.39 (s, 6H), 0.23 (d, *J* = 6.0 Hz, 9H). **Synthesis of 1c.** In a dry flask, potassium carbonate (1.7 g, 12 mmol) and **1b** (1.2 g, 2.0 mmol) were dissolved in a mixture of THF/CH_3_OH (3/2, *v*/*v*; 15 mL). The mixture was stirred at room temperature for 12 h. After evaporating the solvents, the residue was diluted with CH_2_Cl_2_ and washed with brine and water, and the solution was washed with water and then dried over Na_2_SO_4_. The solvent was removed under reduced pressure, and the crude product went through a short pad of silica gel using the CH_2_Cl_2_/CH_3_OH mixture (40/1, *v*/*v*) as the eluent to present the desired product as a light-yellow oil (1.2 g, 80% yield). ^1^H NMR (300 MHz, CDCl_3_, 25 °C): *δ* 6.60 (s, 2H), 4.10 (dd, J = 5.7, 4.0 Hz, 2H), 3.83 (t, J = 5.0 Hz, 2H), 3.67-3.62 (m, 28H), 3.41 (s, 1H), 3.38 (s, 3H), 2.41 (s, 6H). **Synthesis of 1d.** 1,4-dibromo-2,5-diiodobenzene (786 mg, 1.5 mmol), CuI (12 mg, 0.06 mmol), Pd(PPh_3_)_4_ (36 mg, 0.03 mmol) and **1c** (6.3 g, 18.3 mmol) were mixed and dissolved in a THF/Et_3_N mixture (5/2, *v*/*v*, 14 mL), and the solution was stirred at 50 °C for 12 h.. After evaporating the solvents, the residue was diluted with CH_2_Cl_2_, washed with brine and water and then dried over Na_2_SO_4_. The solvent was removed under reduced pressure, and the crude product was purified by silica gel chromatography using a CH_2_Cl_2_/CH_3_OH mixture (20/1, *v*/*v*) as the eluent to give the product a yellow solid crude. The crude was further purified in ether to obtain yellow solid as the product (301 mg, 33.1% yield). ^1^H NMR (300 MHz, CDCl_3_, 25 °C): *δ* 7. 7.74 (s, 2H), 6.65 (s, 4H), 4.15-4.11 (m, 4H), 3.85 (t, J = 4.9 Hz, 4H), 3.74-3.63 (m, 56H), 3.38 (s, 6H), 2.52 (s, 12H). ^13^C NMR (126 MHz, CDCl_3_, rt): 159.10, 142.94, 135.84, 126.74, 123.04, 114.81, 113.38, 95.01, 94.02, 72.05, 70.98, 70.77, 70.75, 70.70, 70.63, 69.79, 67.45, 59.18, 21.72. HRMS (APCI+): *m*/*z* calcd for C_60_H_88_Br_2_O_18_ (M+H^+^), 1257.4337; found 1257.4347.

## 5. Conclusions

This study demonstrates a robust synthetic strategy for constructing amphiphilic helical ladder polymers through the acid-promoted intramolecular cyclization of random-coil precursors. By incorporating optically active binaphthyl units and hydrophilic OEG side chains, the resulting polymers exhibit amplified chiroptical responses, as evidenced by significantly enhanced circular dichroism (CD) and circularly polarized luminescence (CPL) signals. The innovative circularly polarized fluorescence-energy transfer (CPF-ET) system, utilizing these helical polymers as chiral donors to excite achiral coumarin-6 acceptors, achieves green CPL emission with a luminescence dissymmetry factor |g_lum_| value of 2.5 × 10^−4^. These findings not only advance the precision synthesis of chiral ladder polymers but also establish a paradigm for engineering circularly polarized light sources, opening avenues for applications in chiral photonics, asymmetric sensing and energy-efficient optoelectronic devices.

## Data Availability

Data are contained within the article.

## Supplementary Materials

The following supporting information can be downloaded at https://www.mdpi.com/article/10.3390/molecules30122606/s1: Figure S1: IR Spectrum of poly-**1**(*R*) and poly-**2**(*R*); Figure S2: (a) Normalized absorption & PL spectra of poly-**1**(*R*) & poly-**2**(*R*); (b)photo of poly-**2**(*R*) in CH_2_Cl_2_ under UV irradiation (λ_ex_ = 365 nm); Figure S3: Emission spectra of poly-**1**(*R*) and poly-**2**(*R*) in solid state (λ_ex_ = 330 nm); Figure S4: *g*_abs_-absorption spectra of poly-1(*R/S*) (a) and poly-2(*R/S*) (b); Figure S5: CPL- DC spectra of poly-**1**(*R/S*) (a) and poly-**2**(*R/S*) (b) in the solid state; Figure S6: CD and absorbance spectrum of poly-**2**(*R*) with different ratios of C6 (a); Time-resolved fluorescence decay profiles of poly-**2**(*R*) and poly-**2**(*R*) co-assembled with C6 (emission at 460 nm, λ_ex_ = 330 nm) (b); Figure S7: CPL-DC and CD-absorbance spectrum of poly-**2**(*R*) in different ratios of THF and H_2_O; Figure S8: CD-absorbance spectrum of poly-**2**(*R*) with different ratios of C6 (a); Time-resolved fluorescence decay profiles of poly-**2**(*R*) and poly-**2**(*R*) co-assembled with C6 (emission at 460 nm, λ_ex_ = 330 nm) (b); Figure S9 to Figure S15: NMR spectra of the products mentioned in the article.

## Abbreviations

The following abbreviations are used in this manuscript:

CD	Circular Dichroism
CPEL	Circularly Polarized Electroluminescence
CPL	Circularly Polarized Luminescence
DLS	Dynamic Light Scatter
SEM	Scanning Electron Microscope
TADF	Thermally Activated Delayed Fluorescence
TFA	Trifluoroacetic Acid
THF	Tetrahydrofuran
OEG	Oligo(Ethylene Glycol)
FRET	Förster Resonance Energy Transfer
