# Supplementary material for: Synthesis Amphiphilic One-Handed Helical Ladder Polymers with Circularly Polarized Luminescence
Source: Molecules. 2025 Jun 16;30(12):2606. doi: 10.3390/molecules30122606 (PMC12196316; doi:10.3390/molecules30122606)
Supplement: Supplementary file 1 [file molecules-30-02606-s001.zip › molecules-3660939-supplementary.pdf]

molecules

Supplemental Information

# Synthesis Amphiphilic One-Handed Helical Ladder Polymers with Circularly Polarized Luminescence

**Ziheng Pan [1], Wei Zheng ***

Interdisciplinary Materials Research Center, School of Materials Science and Engineering, Tongji University, Shanghai 201804, China; 2230644@tongji.edu.cn
* Correspondence: zhengwei@tongji.edu.cn

## 1. Supporting Data

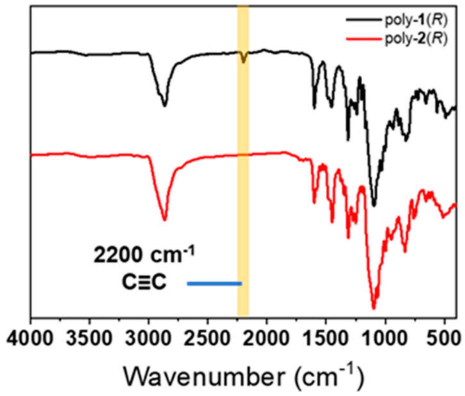


**Figure S1.** IR Spectrum of poly-**1**(*R*) and poly-**2**(*R*).

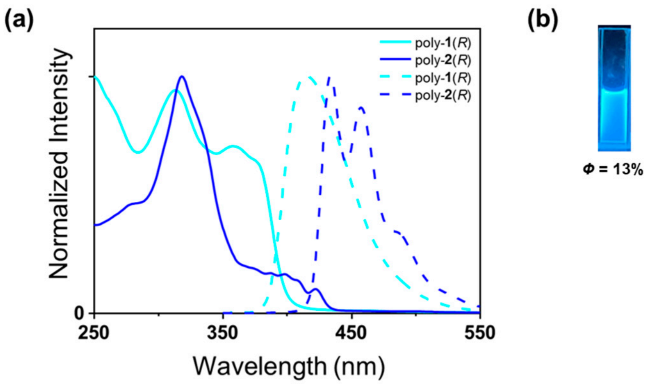


**Figure S2.** (a) Normalized absorption & PL spectra of poly-**1**(*R*) & poly-**2**(*R*); (b)photo of poly-**2**(*R*) in $CH_2Cl_2$ under UV irradiation ($\lambda_{ex}$ = 365 nm).

 https://doi.org/10.3390/molecules30122606

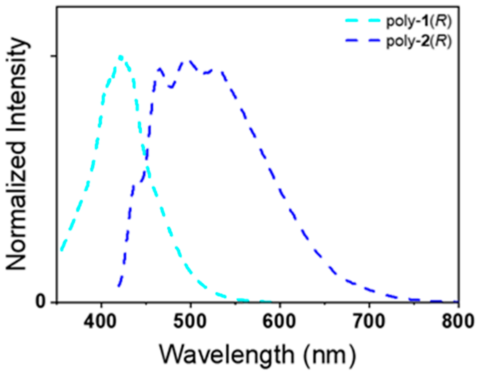


**Figure S3.** PL spectra of poly-**1**(*R*) and poly-**2**(*R*) in solid state ($\lambda_{ex}$ = 330 nm).

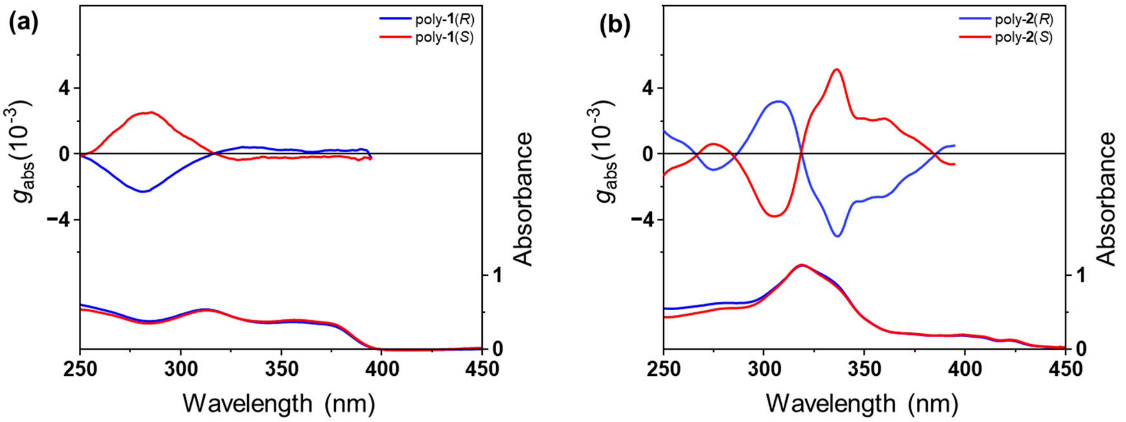


**Figure S4.** $g_{abs}$-absorption spectra of poly-**1**(*R*/*S*) (a) and poly-**2**(*R*/*S*) (b) in $CH_2Cl_2$.

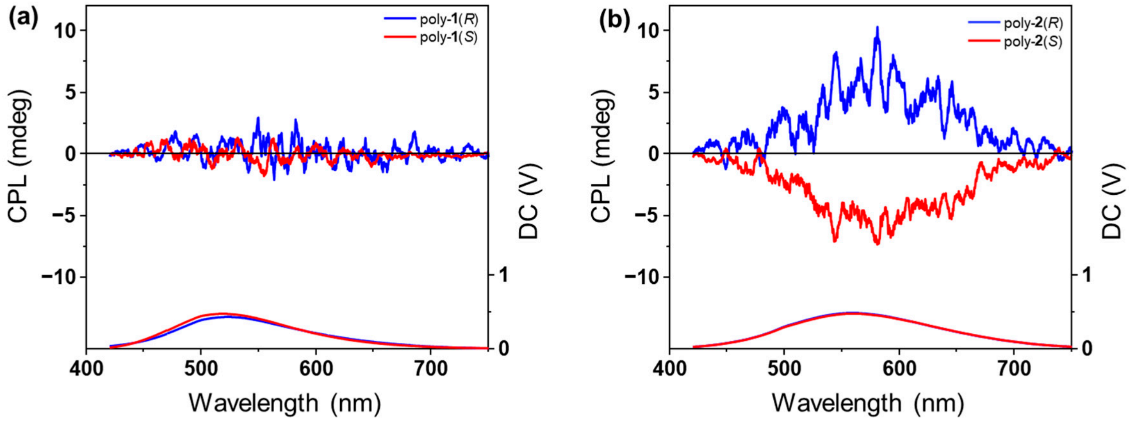


**Figure S5.** CPL-DC spectra of poly-**1**(*R*/*S*) (a) and poly-**2**(*R*/*S*) (b) in solid state.

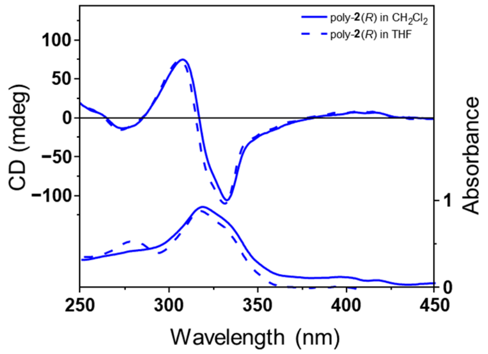

**Figure S6.** CD-absorbance spectrum of poly-**2**(*R*) in THF and $CH_2Cl_2$.

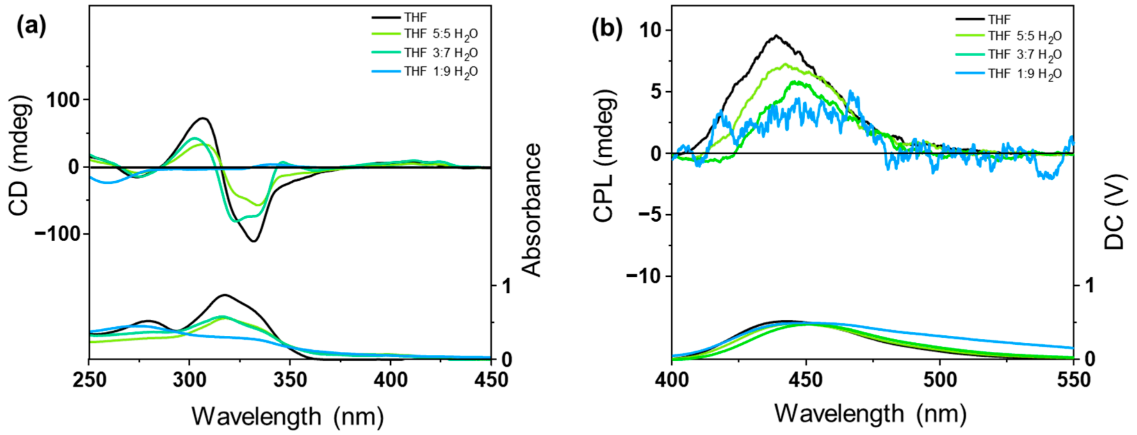


**Figure S7.** CD-absorbance (a) & CPL-DC (b) spectrum of poly-**2**(*R*) in different ratio of THF and $H_2O$.

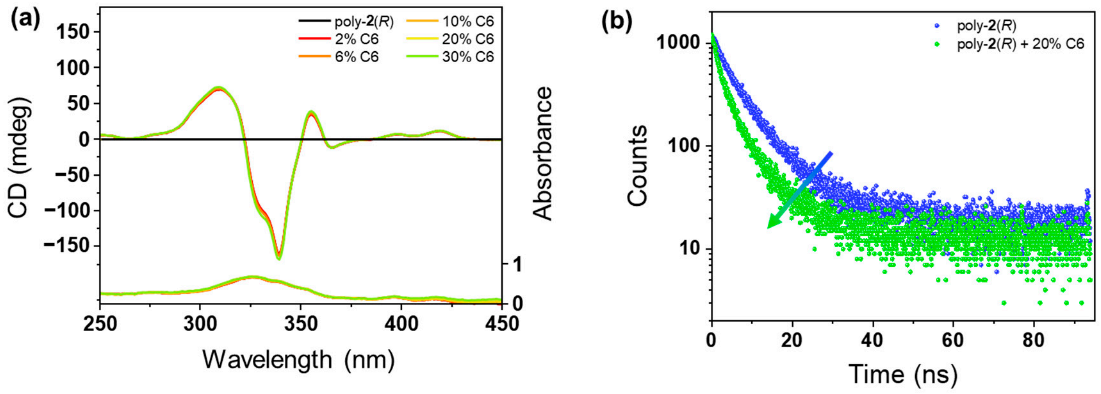


**Figure S8.** CD-absorbance spectrum of poly-**2**(*R*) with different ratio of C6 (a); Time-resolved fluorescence decay profiles of poly-**2**(*R*) & poly-**2**(*R*) co-assembled with C6 (emission at 460 nm, $\lambda_{ex}$ = 330 nm) (b).

## 2 $^1$H and $^{13}$C NMR Spectrum Data

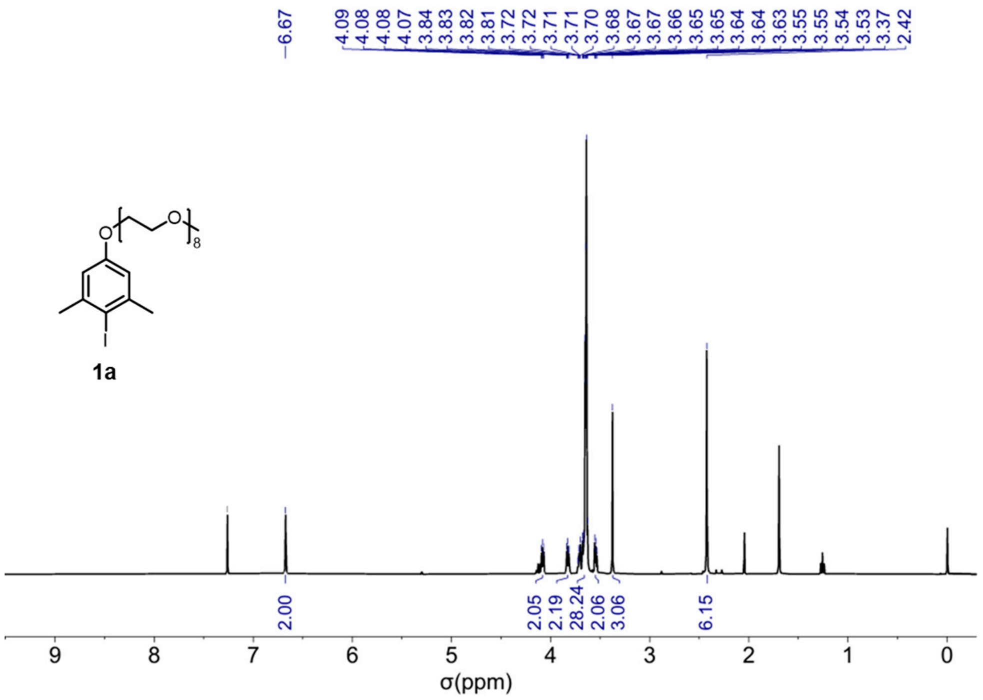


**Figure S9.** $^{1}H$ NMR (300 MHz, $CDCl_3$, 298 K) spectrum of **1a**.

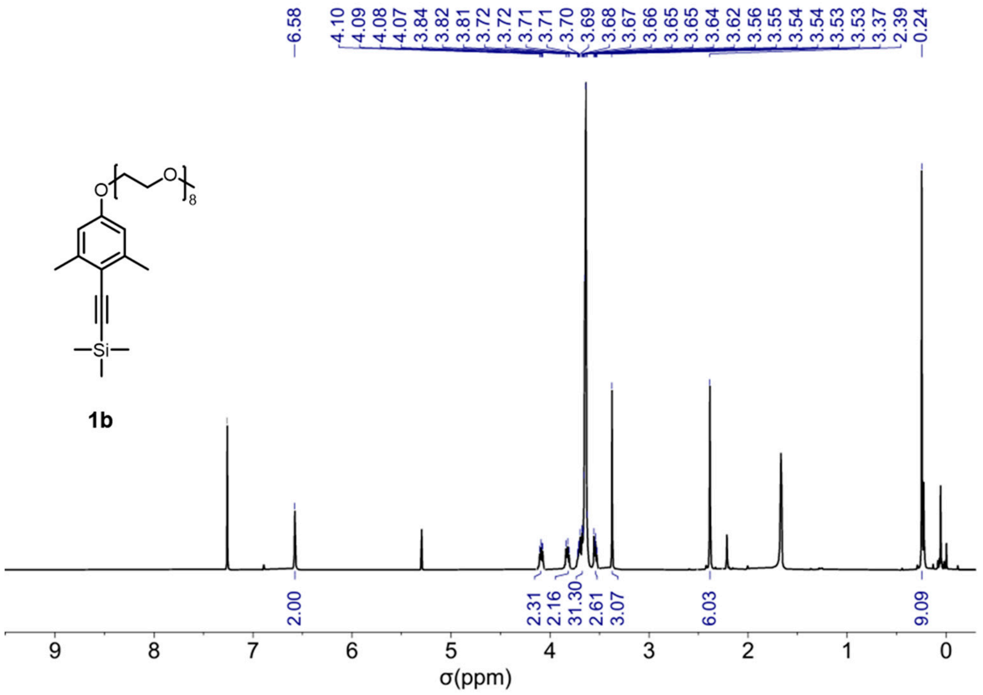


**Figure S10.** $^{1}H$ NMR (300 MHz, $CDCl_3$, 298 K) spectrum of **1b**.

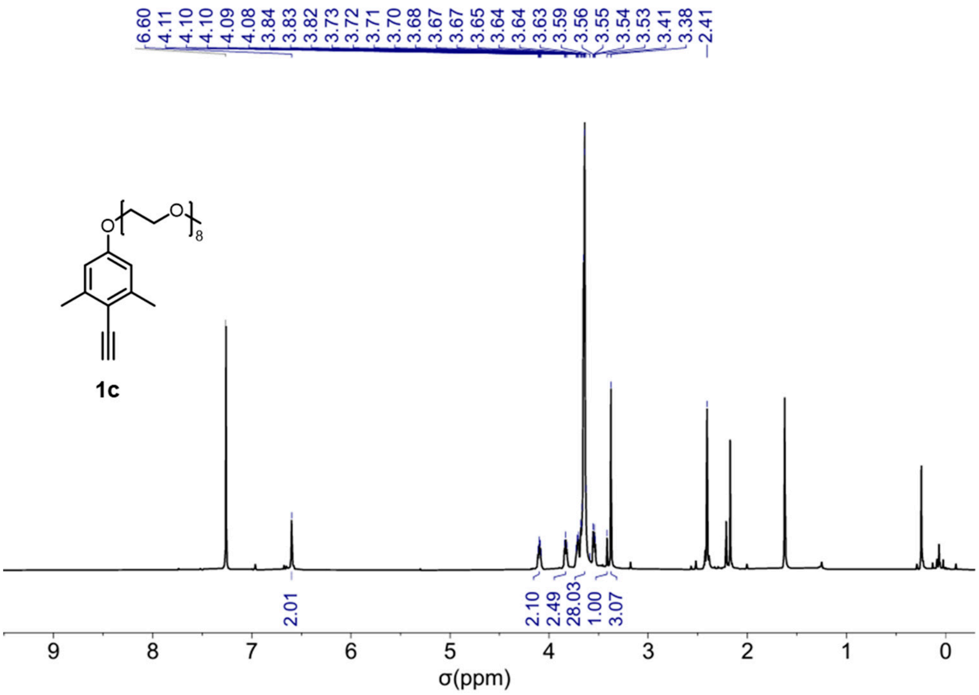

**Figure S11.** $^{1}$H NMR (300 MHz, $CDCl_3$, 298 K) spectrum of **1c.**

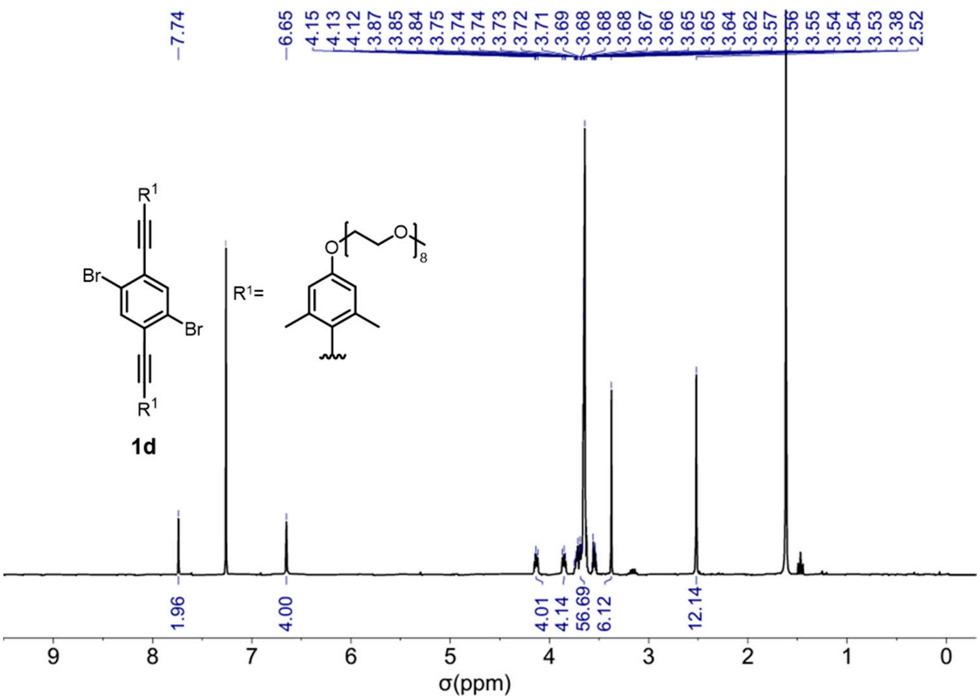

**Figure S12.** $^{1}$H (300 MHz, $CDCl_3$, 298 K) NMR spectrum of **1d.**

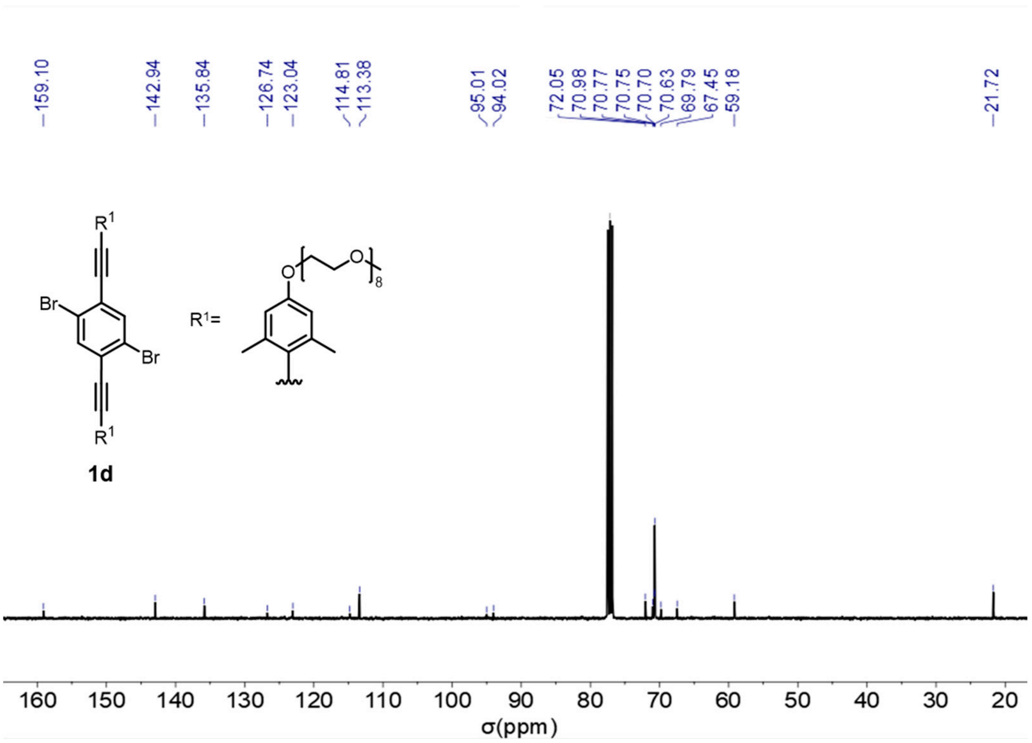


**Figure S13.** $^{13}$C (101 MHz, $CDCl_3$, 298 K) NMR spectrum of **1d.**

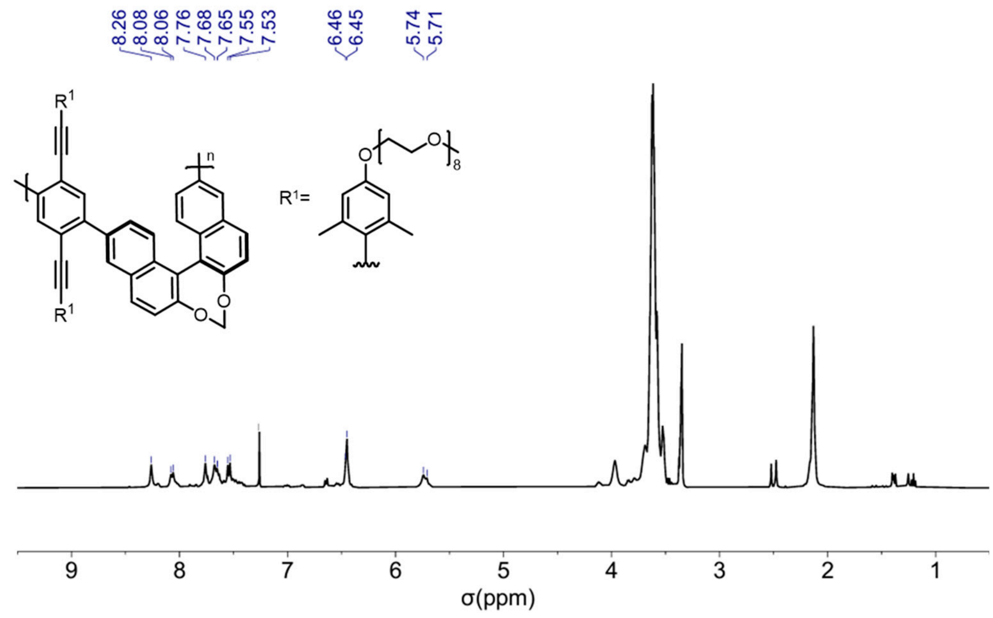


**Figure S14.** $^{1}$H NMR (400 MHz, $CDCl_3$, 298 K) spectrum of poly-**1**(*R*).

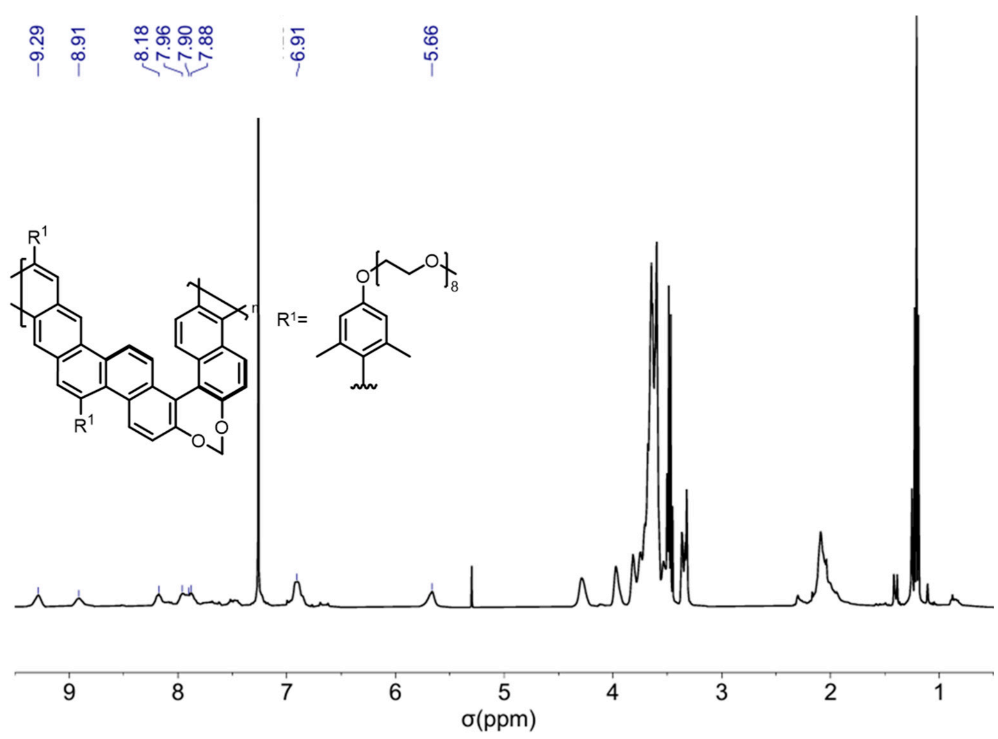


**Figure S15.** $^1$H NMR (400 MHz, $CDCl_3$, 298 K) spectrum of poly-**2**(*R*).
